# Characterization of the Kaposi’s sarcoma-associated herpesvirus terminase complex component ORF29

**DOI:** 10.1128/spectrum.03308-24

**Published:** 2025-09-09

**Authors:** Yuki Iwaisako, Karin Shimizu, Youichi Suzuki, Takashi Nakano, Masahiro Fujimuro

**Affiliations:** 1 Department of Cell Biology, Kyoto Pharmaceutical University 12916, Kyoto, Japan; 2 Department of Microbiology and Infection Control, Faculty of Medicine, Osaka Medical and Pharmaceutical University 13010, Osaka, Japan; University of Florida College of Dentistry, Gainesville, Florida, USA

**Keywords:** Kaposi's sarcoma-associated herpesvirus, human herpesviruses 8, terminase, lytic replication, capsid formation, DNA replication, viral-DNA processing, ORF29

## Abstract

Kaposi’s sarcoma-associated herpesvirus (KSHV) belongs to the Gammaherpesvirinae subfamily. During the lytic phase of herpesviruses, viral capsids form in the host cell nucleus, and the replicated viral genome is packaged into these capsids. The herpesviral genome is replicated as a precursor head-to-tail concatemer consisting of tandemly repeated genomic units, each flanked by terminal repeats (TRs). The herpesvirus terminase complex packages a single genomic unit into a capsid by cleaving the TRs in the precursor genome. Although the terminase complexes of alpha- and beta-herpesviruses are well characterized, the KSHV terminase complex is poorly understood. KSHV ORF7, ORF67.5, and ORF29 are thought to be components of this complex. We previously reported that KSHV deficient in either ORF7 or ORF67.5 formed immature, soccer ball-like capsids and failed to cleave the TRs, resulting in decreased virion production. Moreover, ORF7 interacted with both ORF29 and ORF67.5; however, ORF29 and ORF67.5 did not interact with each other. Thus, although ORF7 and ORF67.5 are important for KSHV terminase function, the function of ORF29 remains largely unknown. In this study, we constructed an ORF29-deficient KSHV and analyzed its virological properties. ORF29 was found to be essential for virion production and TR cleavage. Numerous immature, soccer ball-like capsids were observed in cells harboring ORF29-deficient KSHV. The N-terminal region of ORF29 was important for its interaction with ORF7, although the full-length ORF29 was required for effective assembly of the KSHV terminase complex. Furthermore, ORF29 preferentially interacted with itself rather than with ORF7. Thus, our data show that ORF29 functions as a fundamental component of the terminase complex.

**IMPORTANCE:**

Because the role of ORF29 in the Kaposi’s sarcoma-associated herpesvirus (KSHV) terminase complex remains unknown, we constructed ORF29-deficient KSHV. Our results demonstrated that ORF29 functions as a component of the KSHV terminase and is essential for mature capsid formation, terminal repeat (TR) cleavage, and terminase complex assembly. Moreover, ORF29 strongly interacted with itself. In herpes simplex virus 1 (HSV-1), the terminase complex (comprising UL15, UL28, and UL33) forms a trimer, and six such trimers assemble into a hexameric ring. The HSV-1 genome passes through this ring and undergoes TR cleavage and genome packaging into a capsid. The self-interaction of ORF29 may be involved in the multimerization of the terminase complex or in the formation of the KSHV terminase ring.

## INTRODUCTION

Kaposi’s sarcoma-associated herpesvirus (KSHV), also known as human herpesvirus 8, causes Kaposi’s sarcoma and primary effusion lymphoma in AIDS patients (1). When KSHV infects healthy individuals, it establishes a latent infection in vascular endothelial cells or B cells. In the latent infection state, KSHV latent genes are expressed, and these gene products contribute to cell proliferation, inhibition of apoptosis, stabilization of the viral genome, and maintenance of KSHV latency. In individuals latently infected with KSHV, reactivation can be triggered by UV exposure, immunodeficiency, drug treatment, or hormonal changes. Reactivation of KSHV initiates a transition to the lytic phase, which is characterized by the production of progeny virus (2). In the KSHV lytic phase, lytic gene expression, viral genome replication, capsid assembly, viral particle formation, and budding occur in sequence. KSHV lytic genes are classified as immediate-early (IE), delayed-early (DE), and late (L) genes, based on their expression timing and requirements (2). The mechanisms of precursor genome synthesis, precursor genome processing, genome insertion into the capsid, capsid maturation, and formation of infectious virus particles (i.e., virions) remain largely unknown. However, these mechanisms have been proposed to include the following steps. Initially, the KSHV DNA genome is replicated as head-to-tail concatemers. A head-to-tail concatemer consists of tandemly repeated genomic units in linear form, with each unit flanked by terminal repeat sequences (TRs) (3). A single TR comprises a G/C-rich sequence of 801 base pairs (bps), and a TR set contains 20–40 tandemly linked repeats. The unit-length viral genome is flanked by a set of 20–40 TRs. It is proposed that the viral genome is packaged into a capsid and that a unit-length genome is generated by cleavage at the TR region of the precursor genome. The order of genome packaging and cleavage is unclear, but these processes are mediated by the viral terminase complex. However, little is known about how the KSHV precursor genome is processed and inserted into a capsid by the terminase complex. These processes result in the formation of a mature capsid, which then acquires viral tegument proteins and a lipid bilayer envelope, ultimately egressing from the host cell as virions.

Unlike the terminase complex of KSHV, that of herpes simplex virus 1 (HSV-1) is better understood. The HSV-1 terminase complex is responsible for packaging the viral genome into the capsid lumen, cleaving the TR region of the viral genome precursor, and producing unit-length viral genomes. Furthermore, the HSV-1 terminase complex exhibits motor activity that moves the DNA strand in an ATP-dependent manner to facilitate genome packaging into the capsid. The HSV-1 terminase complex also recognizes a specific DNA sequence for TR cleavage (4). In contrast, only a few studies have been published on the terminase complex of KSHV. Based on homology with other herpesviruses, KSHV ORF7, ORF29, and ORF67.5 are considered components of the terminase complex (5). We have previously reported that ORF7 and ORF67.5 are required for virus production and are essential for TR cleavage (6–8). KSHV lacking ORF7 or ORF67.5 failed to form mature capsids and instead formed soccer ball-like capsids, which are thought to be immature capsids (6–8). However, the function of ORF29 as a component of the KSHV terminase complex remains unknown. Therefore, in this paper, we focused on ORF29 and characterized its role within the KSHV terminase complex.

The terminase functions of KSHV ORF29 remain largely unclear, despite the HSV-1 homolog of ORF29 (UL15) being well understood. The coding region of the HSV-1 UL15 gene consists of a first exon and a second exon separated by a single intron. UL15 mRNA is generated by splicing reactions that remove the central intron (9–11). At the non-permissive temperature (NPT), the UL15 temperature-sensitive (ts) mutant could replicate the viral genome but was unable to package it into the capsid (12). Furthermore, concatemeric viral genomes accumulated in UL15 ts mutant-infected cells at the NPT (13). Roizman et al. reported that in HSV-1 (F)-infected cells, two types of UL15 proteins [with molecular weights (Mws) of 35 and 75 kDa] shared the C-terminus encoded by the second exon of UL15 (13). The 75 kDa UL15 protein was required for viral genome cleavage and packaging, and HSV-1 mutants with genetic disruption of UL15 failed to form mature C-capsids, instead forming immature B-capsids (14). Because a 35 kDa protein is detected in HSV-1 mutant-infected cells with stop codons inserted into the first exon of UL15, this indicates that the 35 kDa protein is the translation product of the second exon of UL15 (15). This product is referred to as UL15.5. UL15.5 is not required for HSV-1 infectious virus production, but its function remains unknown (16). Baines et al. also reported that full-length UL15 and two low-Mw forms of UL15 (3 kDa- and 4 kDa-shortened forms) were detected in purified B-capsids (17, 18). Taken together, these findings indicate that UL15 plays a role in both viral genome cleavage and packaging into the capsid. Moreover, the UL15 gene expresses multiple proteins with different Mws.

The KSHV ORF29 gene encodes a 687-amino acid (aa) protein. This gene contains one intron between the first and second exons (19, 20). Le Grice et al. found that the C-terminal domain of ORF29 has DNA sequence-independent nuclease activity (21). We demonstrated that ORF29 interacts with ORF7 but not with ORF67.5 (6). Moreover, the interaction between ORF7 and ORF67.5 is enhanced by ORF29, and the interaction between ORF29 and ORF7 is enhanced by ORF67.5 (8). Glaunsinger et al. generated an ORF29.stop virus in which Leu338 and Gln339 of KSHV ORF29 are mutated to stop codons. Production of infectious virus from the ORF29.stop mutant was impaired, and expression of the L gene (K8.1) was decreased at both the mRNA and protein levels. Additionally, viral genome replication was also reduced in ORF29.stop KSHV (22). Although several functions of ORF29 have been reported, its contribution to KSHV terminase function remains unresolved.

In this paper, we attempted to characterize ORF29 by generating KSHV lacking full-length ORF29 (ORF29-deficient KSHV) and its revertant. ORF29-deficient KSHV was impaired in virus production and infectious virion formation, but interestingly, neither K8.1 expression nor viral genome replication was affected. ORF29-deficient KSHV failed to form mature capsids and instead formed immature, soccer ball-like capsids. It also lost the ability to cleave TRs. Furthermore, we found that ORF29 preferentially interacted with itself rather than with ORF7.

## RESULTS

### Construction of an ORF29-deficient KSHV bacterial artificial chromosome

**Fig 1:**
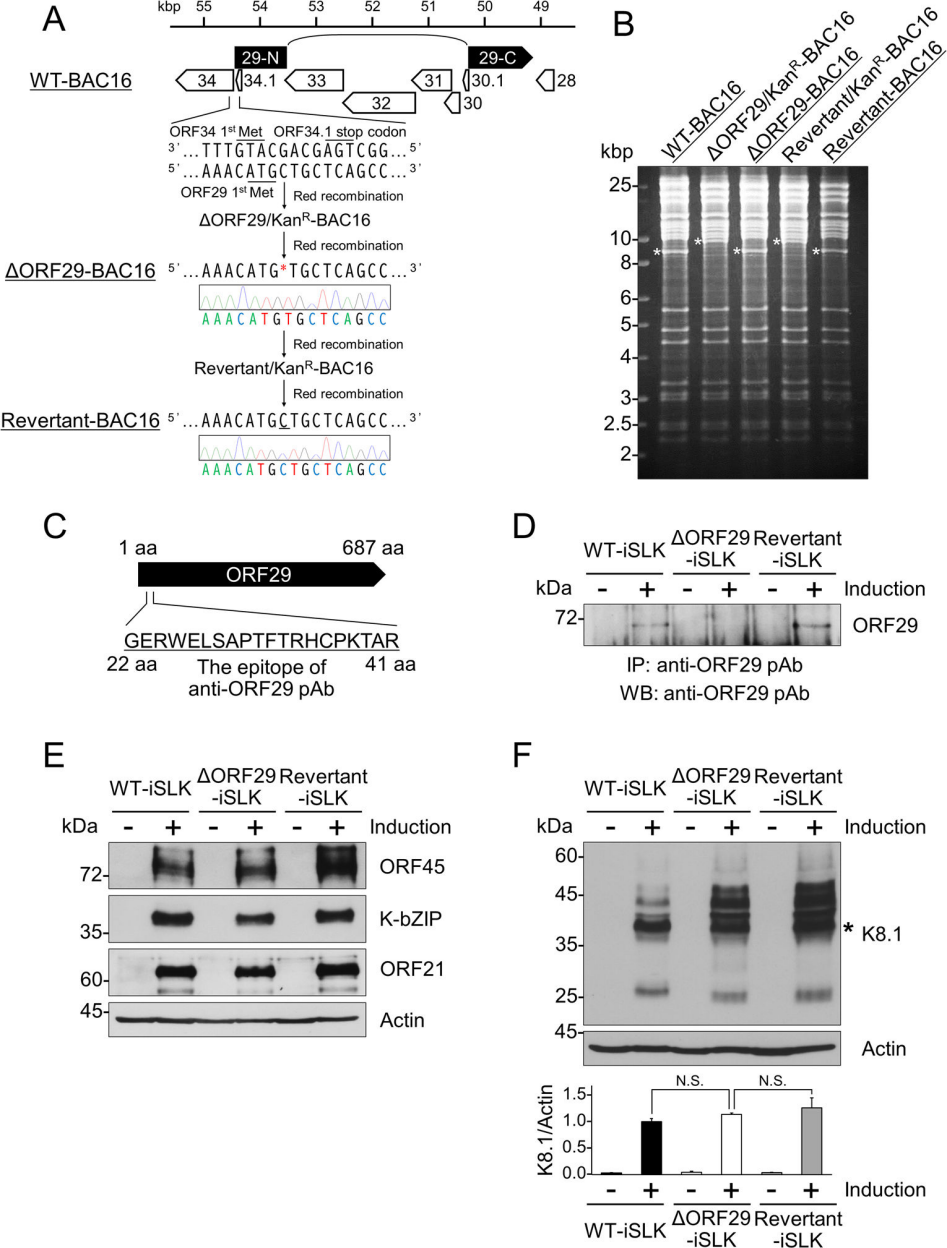
Construction of ΔORF29-BAC16 and Revertant-BAC16. (**A**) Conceptual diagram showing the location of KSHV ORF29 (nucleotides 49,179–50,321 and 53,572–54,492; accession number: GQ994935) and the process used to generate each mutant KSHV-BAC. The nucleotide sequences adjacent to each mutagenesis site were confirmed by Sanger sequencing. (**B**) Each BAC DNA was digested with EcoRI, and the resulting DNA fragments were separated by agarose gel electrophoresis to confirm the insertion and removal of the kanamycin resistance cassette. Asterisks indicate the insertion or deletion of the kanamycin resistance cassette in each BAC clone. (**C**) The antigenic peptide sequence used for the preparation of the rabbit anti-ORF29 pAb; aa, amino acids. (**D**) Validation of endogenous ORF29 protein expression in lytic-induced (+) or uninduced (−) WT-iSLK, ΔORF29-iSLK, and Revertant-iSLK cells. WT-BAC16, ΔORF29-BAC16, and Revertant-BAC16 were transfected into iSLK cells to establish stable BAC16-harboring iSLK cell lines (designated WT-iSLK, ΔORF29-iSLK, and Revertant-iSLK cells, respectively). Each cell line was treated or untreated with doxycycline (Dox) and sodium butyrate (SB) for 72 h to induce lytic reactivation. Cells were then lysed with RadioImmunoPrecipitation Assay (RIPA) buffer. Cell lysates were subjected to immunoprecipitation (IP) using anti-ORF29 pAb-conjugated Dynabeads coupled to Protein G. The immunoprecipitates were analyzed by WB with anti-ORF29 pAb. (**E**) Protein expression of lytic genes in lytic-induced (+) or uninduced (−) WT-iSLK, ΔORF29-iSLK, and Revertant-iSLK cells. Each cell line was treated or untreated with Dox and SB for 72 h and analyzed by WB using the indicated antibodies (Abs). Actin was used as a loading control. (**F**) K8.1 protein expression in each iSLK cell line. Cells were treated with Dox and SB for 72 h and subjected to WB. Signals marked with an asterisk were quantified and normalized to the actin signal. Values from Dox- and SB-treated WT-iSLK cells were defined as 1.0. N.S., not statistically significant (*P* > 0.05).

To analyze the function of KSHV ORF29, we constructed a full-length ORF29-deficient KSHV bacterial artificial chromosome (BAC) clone (ΔORF29-BAC16) and its reverse-mutated KSHV BAC clone (Revertant-BAC16). We generated ΔORF29-BAC16 by introducing a frameshift via deletion of a C–G bp located 1 bp downstream of the ORF29 start codon (ATG) (Fig. 1A). Although ORF34 and ORF34.1 are near the ORF29 start codon in the KSHV genome, the mutation site in ΔORF29-BAC16 does not overlap with the ORF34 or ORF34.1 coding regions. The generated ΔORF29-BAC16 clone contains nonsense mutations in the ORF29 region. Thus, ΔORF29-BAC16 encodes 25 amino acids unrelated to ORF29 from the start codon to 75 bp within ORF29, followed by a stop codon at positions 76–78 bp. Furthermore, Revertant-BAC16 was generated by reinserting the missing 1 bp (i.e., the C–G bp) into ΔORF29-BAC16 (Fig. 1A). The nucleotide sequences around the mutagenesis sites in ΔORF29-BAC16 and Revertant-BAC16 were confirmed by Sanger sequencing (Fig. 1A). Each BAC clone was digested with the restriction enzyme EcoRI, and the insertion and removal of the kanamycin resistance gene during mutagenesis were confirmed by changes in band patterns on agarose gel electrophoresis (Fig. 1B). Wild-type (WT)-BAC16, ΔORF29-BAC16, and Revertant-BAC16 were transfected into iSLK cells and selected with hygromycin B to establish cells stably harboring BAC16, defined as WT-iSLK cells, ΔORF29-iSLK cells, and Revertant-iSLK cells, respectively. When iSLK cells harboring BAC16 (i.e., KSHV latently infected cells) are treated with doxycycline (Dox) and sodium butyrate (SB), lytic reactivation is effectively induced via Dox-elicited replication and transcription activator expression (23). Next, we attempted to confirm the loss of ORF29 expression in ΔORF29-iSLK cells and restoration of ORF29 expression in Revertant-iSLK cells by western blotting (WB) using an antibody (Ab) specific for ORF29. A rabbit anti-ORF29 polyclonal antibody (pAb) was raised using the 22 to 41 aa region (GERWELSAPTFTRHCPKTAR) within ORF29 as a peptide antigen (Fig. 1C). Each cell line was treated (or untreated) with Dox and SB for 72 h to induce lytic reactivation. We attempted to detect endogenous ORF29 by WB but were unable to do so due to numerous nonspecific signals (data not shown). Therefore, the ORF29 protein was immunoprecipitated with anti-ORF29 pAb, and the precipitates were subjected to WB. The calculated Mw of endogenous ORF29 is 76.5 kDa. When lytic reactivation was induced, ORF29 expression was detected in WT-iSLK and Revertant-iSLK cells but not in ΔORF29-iSLK cells (Fig. 1D). In all cell lines, ORF29 was not detected in the non-lytic state (Fig. 1D).

In addition to ORF29, the expression of other lytic proteins in each iSLK cell line was examined. The expression of the IE protein ORF45, the DE protein K-bZIP, the L protein K8.1, and ORF21 was detected in WT-iSLK, ΔORF29-iSLK, and Revertant-iSLK cells under lytic conditions (Fig. 1E and F). These results indicate that deficiency of the ORF29 protein or gene in lytic-induced KSHV-harboring cells does not remarkably affect the expression of ORF45, K-bZIP, ORF21, or K8.1 proteins.

### Virion production is impaired in ORF29-deficient KSHV

**Fig 2:**
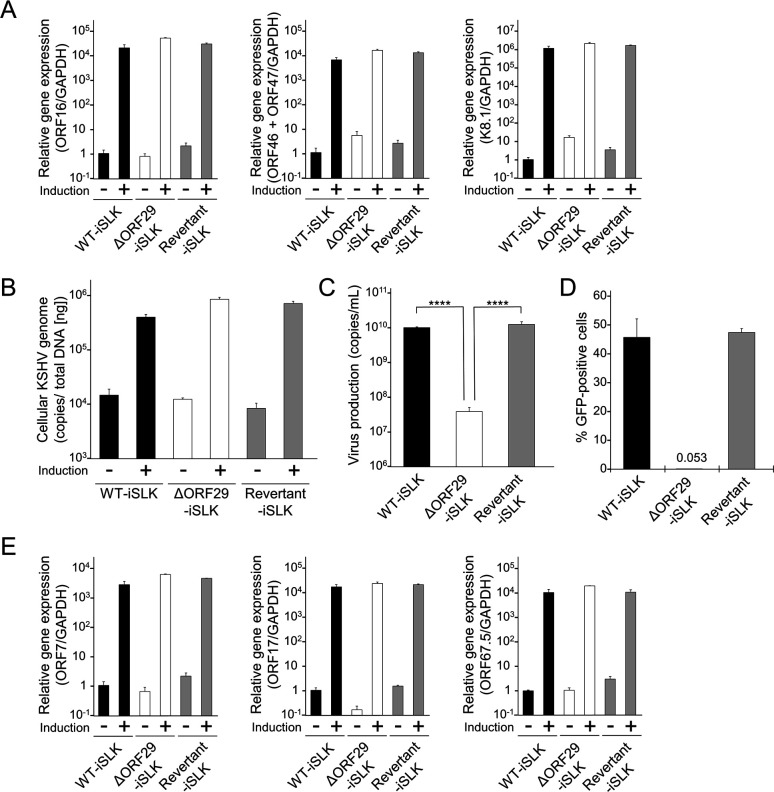
Characterization of ΔORF29 KSHV. (**A**) Transcription of KSHV lytic genes in lytic-induced (+) or uninduced (−) WT-iSLK, ΔORF29-iSLK, and Revertant-iSLK cells. Each cell line was treated or untreated with Dox and SB for 72 h to induce lytic reactivation. Total RNA was extracted and subjected to RT-qPCR to evaluate mRNA expression of the following lytic genes: an immediate-early (IE) gene ORF16, delayed-early (DE) genes ORF46 and ORF47, and a late (L) gene K8.1. Viral gene mRNA levels were normalized to GAPDH mRNA levels. Values from uninduced WT-iSLK cells were defined as 1.0. (**B**) Intracellular KSHV genome copy number. Each cell line was treated or untreated with Dox and SB, followed by intracellular DNA extraction. KSHV genome copy number was quantified by qPCR and normalized to total DNA content. (**C**) Extracellular encapsidated KSHV genome copy number. Cells were treated with Dox and SB for 72 h, and culture supernatants were collected. Encapsidated viral genomes were purified from DNase-treated supernatants, and viral genome copy numbers were quantified by qPCR. ****, *P* < 0.001. (**D**) Production of infectious virions. Each cell line was treated with Dox and SB for 72 h, and culture supernatants were used in a supernatant transfer assay. Fresh HEK293T cells were infected with the supernatants, and at 24 h post-infection, the percentage of GFP-positive HEK293T cells was measured by flow cytometry to evaluate infectious virus production. (**E**) Each cell line was treated or untreated with Dox and SB for 72 h, and total RNA was extracted and subjected to RT-qPCR to evaluate mRNA expression of ORF7, ORF17, and ORF67.5. Viral gene mRNA levels were normalized to GAPDH mRNA levels. Values from uninduced WT-iSLK cells were defined as 1.0.

To assess the contribution of ORF29 to virus production, we evaluated the transcription of lytic genes, the amount of intracellular viral genome replication, and virus production in lytic-induced ΔORF29-iSLK cells. WT-iSLK, ΔORF29-iSLK, and Revertant-iSLK cells were treated with Dox and SB for 72 h, and the cells and culture supernatants were collected. The mRNA expression levels of the IE gene ORF16, the DE genes ORF46 and ORF47, and the L gene K8.1 were measured by reverse transcription-quantitative PCR (RT-qPCR) using total RNA from harvested cells. There were no remarkable differences in the expression levels of the tested lytic genes in each cell line (Fig. 2A). The intracellular viral genome copy number from harvested cells was quantified by qPCR. The amount of intracellular viral genome was comparable across all cell lines (Fig. 2B). Next, extracellular encapsidated viral genome copy number was evaluated by quantifying DNase-treated culture supernatants using qPCR. The encapsidated viral copy number in the ΔORF29-iSLK cell supernatant was significantly decreased compared to WT-iSLK and Revertant-iSLK cell supernatants (Fig. 2C). Next, infectious virus production from ΔORF29-iSLK cells was examined by a supernatant transfer assay. Culture supernatants from each lytic-induced iSLK cell line were co-cultured with fresh HEK293T cells, and green fluorescent protein (GFP) positivity in HEK293T cells was measured by flow cytometry. Because BAC16 contains the GFP gene, the infectivity of BAC16-derived KSHV can be assessed by measuring GFP fluorescence (24). The amount of infectious virus produced by ΔORF29-iSLK cells was markedly reduced compared to WT-iSLK and Revertant-iSLK cells (Fig. 2D). Additionally, we examined the mRNA expression levels of ORF7, ORF67.5, and ORF17 in lytic-induced ΔORF29-iSLK cells. ORF7 and ORF67.5 are components of the terminase, and ORF17 is involved in mature capsid formation. As expected, ORF29 deficiency had no effect on their expression (Fig. 2E). These results indicate that ORF29 is not essential for KSHV lytic gene expression or viral genome replication but is important for the production of infectious virions.

**Fig 3:**
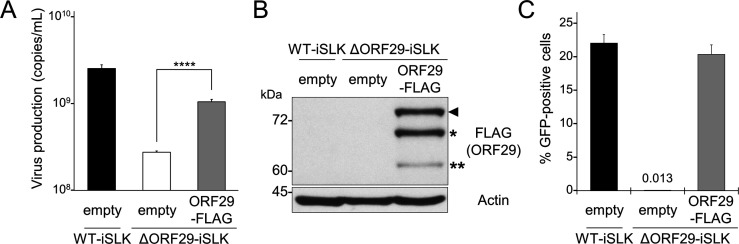
Complementation of reduced virion production in ORF29-deficient KSHV by exogenous ORF29 expression. (**A**) Transient expression of exogenous ORF29 restored the reduction in extracellular encapsidated viral genomes observed in ΔORF29-iSLK cells. ΔORF29-iSLK cells were transiently transfected with the ORF29-FLAG plasmid or a control plasmid lacking the ORF29 gene (empty vector). Cells were then treated with Dox and SB for 72 h to induce lytic reactivation. Encapsidated viral genomes in the culture supernatant were quantified by qPCR. ****, *P* < 0.001. (**B**) Exogenous ORF29 expression in the samples described in (**A**) was confirmed by WB using anti-FLAG Ab. The black arrowhead indicates ORF29-FLAG. The single asterisk (*) indicates the translation product from the second methionine (M42) of ORF29, and the double asterisk (**) indicates the translation product from the third methionine (M105) of ORF29. Note: we confirmed that the translation is initiated not only from the first AUG, but also from the second and third AUGs within ORF29 mRNA in the exogenous expression of the ORF29 plasmid (data not shown). (**C**) Transient expression of exogenous ORF29 rescued the decrease in infectious virion production in ΔORF29-iSLK cells. ΔORF29-iSLK cells were transiently transfected with the ORF29-FLAG plasmid or control plasmid (empty vector), then treated with Dox and SB for 72 h. Harvested culture supernatants were analyzed by a supernatant transfer assay. Fresh HEK293T cells were infected with the produced virions, and at 24 h post-infection, the percentage of GFP-positive HEK293T cells was measured by flow cytometry.

To further examine the contribution of ORF29 to virion production, we performed a complementation assay in ΔORF29-iSLK cells. The extracellular encapsidated viral genome copy number, reduced in ΔORF29-iSLK cells compared to WT-iSLK cells, was significantly restored by transient transfection of a C-terminal FLAG-tagged ORF29 (ORF29-FLAG) expression plasmid (Fig. 3A). Expression of exogenous ORF29-FLAG was confirmed by WB (Fig. 3B). Furthermore, the ability of ΔORF29-iSLK cells to produce infectious virus, which was inhibited due to the loss of ORF29, was rescued by transfection of the ORF29-FLAG plasmid (Fig. 3C). These results confirm that ORF29 is required for virion production.

### ORF29-deficient KSHV capsid maturation is arrested at the immature soccer ball-like capsid stage

**Fig 4:**
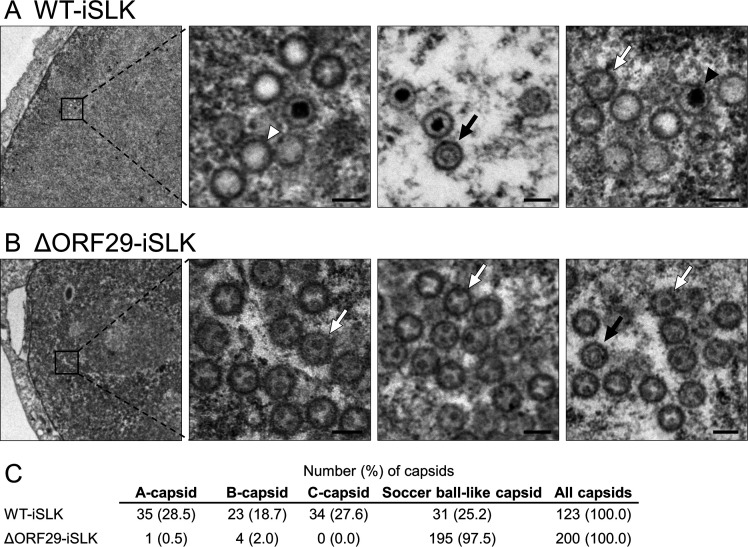
The soccer ball-like capsids were mainly produced in lytic-induced ΔORF29-iSLK cells. Transmission electron microscopy (TEM) images showing the morphology of capsids formed in the nuclei of (**A**) WT-iSLK cells and (**B**) ΔORF29-iSLK cells during the lytic phase. Cells were treated with Dox and SB for 48 h to induce the lytic phase, and nuclear capsids were observed by TEM. Black arrowheads, white arrowheads, black arrows, and white arrows indicate C-capsids, A-capsids, B-capsids, and soccer ball-like capsids, respectively. Scale bars, 100  nm. (**C**) Quantification of each type of capsid observed in lytic-induced WT-iSLK and ΔORF29-iSLK cells.

The following three types of KSHV capsid structures have been defined from electron microscopic images: A-capsids, which are empty capsids; B-capsids, which contain a globular scaffold protein but lack the viral genome; and C-capsids, which are mature capsids containing the viral genome but lack the scaffold protein (25). We previously reported that KSHVs deficient in ORF7 or ORF67.5, which are components of the KSHV terminase complex, fail to form mature capsids and instead form immature soccer ball-like capsids (7, 8). To investigate the contribution of ORF29 to capsid maturation, the morphology of capsids formed in WT-iSLK and ΔORF29-iSLK cell lines was observed by transmission electron microscopy. Cells were treated with Dox and SB for 48 h, and the nuclear capsids were photographed. In WT-iSLK cells, A-capsids, B-capsids, C-capsids, and soccer ball-like capsids were detected (Fig. 4A). In contrast, C-capsids were not observed in ΔORF29-iSLK cells, where most capsids formed were soccer ball-like capsids (Fig. 4B). Figure 4C shows the number of capsids of each type. These data indicate that KSHV capsid formation was arrested at the immature soccer ball-like capsid stage in lytic-induced ΔORF29-iSLK cells. Thus, similar to ORF7 and ORF67.5, ORF29 is important for KSHV capsid maturation.

### ORF29 is essential for cleavage of the TRs in the KSHV genome

**Fig 5:**
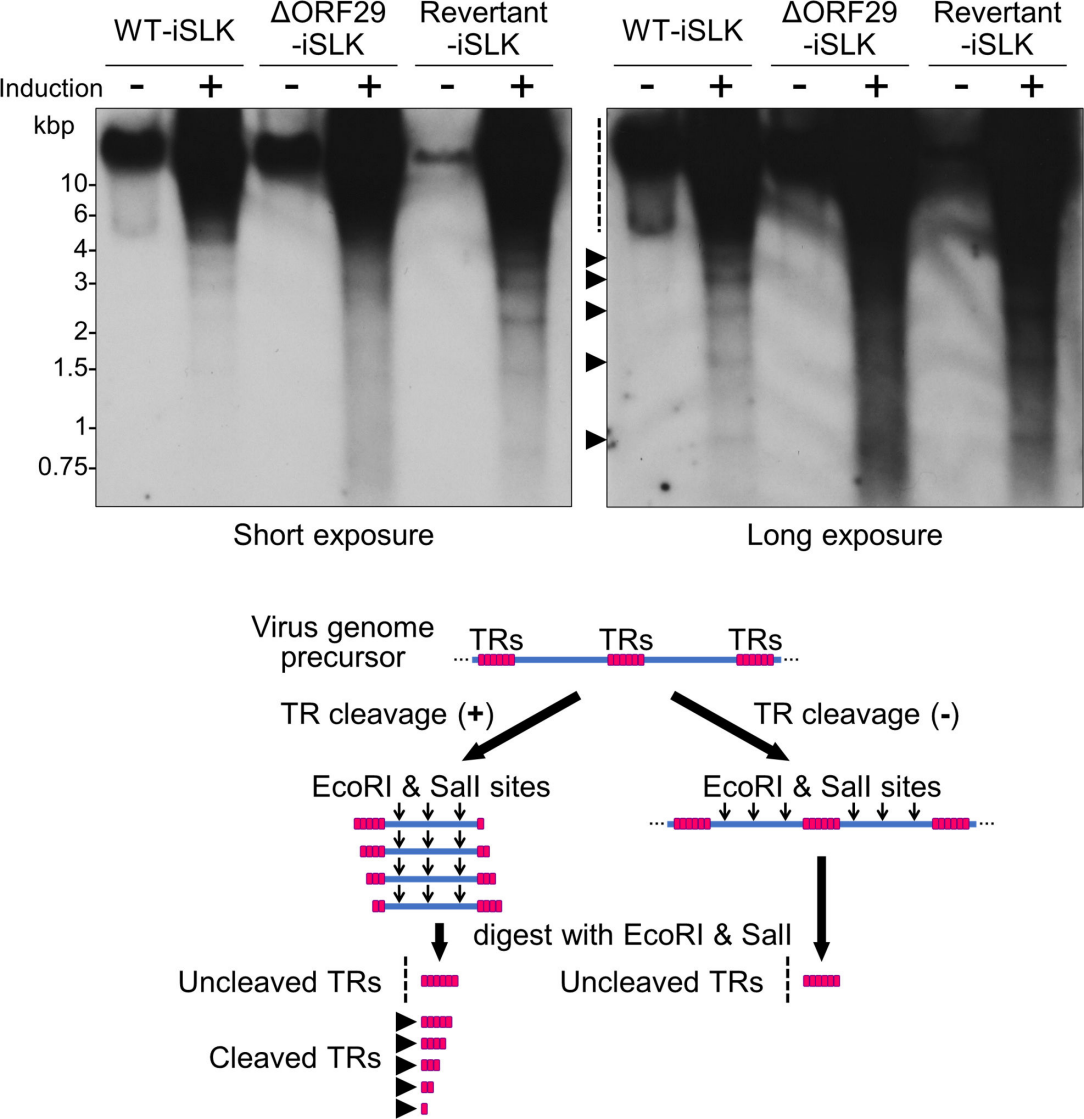
ORF29-deficient KSHV failed to cleave the terminal repeats (TRs) in the viral genome precursor. WT-iSLK, ΔORF29-iSLK, and Revertant-iSLK cells were treated or untreated with Dox and SB for 72 h to induce lytic reactivation. Intracellular genomic DNA was purified from lytic-induced (+) or uninduced (−) cells. The genomic DNA was digested with EcoRI and SalI, then subjected to Southern blotting. Uncleaved and cleaved TRs from the KSHV genome were detected using a digoxigenin-labeled 1× TR probe. The left panel shows a short exposure; the right panel shows a long exposure. The dotted line indicates uncleaved TRs, and black arrowheads indicate cleaved TRs. The cartoon shows the principle of TR cleavage detection. KSHV genomes replicate as linear precursors containing tandemly repeating units connected by TR regions. The terminase complex cleaves the DNA at the TR sites within the replicated genome. Purified viral DNA was digested with EcoRI and SalI. These restriction enzymes can digest the viral ORFs, but not the TRs. The digested DNA was analyzed by Southern blotting using 1× TR as a probe (7, 8).

The terminase complex encapsidates the single unit-length viral genome into the capsid and cleaves the TR of the viral genome precursor, resulting in the formation of a mature capsid. Proper cleavage at the TR site within the viral genome precursor by the terminase complex can be assessed by Southern blotting using a probe targeting 1× TR (7, 8, 26). To analyze the contribution of ORF29 to terminase genome cleavage activity, TR cleavage in ΔORF29-iSLK cells was compared to WT-iSLK cells. Total DNA from lytic-induced iSLK cells was digested with EcoRI and SalI to release the TRs, which were detected by Southern blotting. Cleaved TRs were not detected in WT-iSLK, ΔORF29-iSLK, or Revertant-iSLK cells under non-lytic conditions. When lytic reactivation was induced, cleaved TRs were detected in WT-iSLK and Revertant-iSLK cells, but not in ΔORF29-iSLK cells (Fig. 5). These results indicate that ORF29 is required for TR cleavage by the KSHV terminase complex. Additionally, the amount of uncleaved TRs increased after lytic induction in all cell lines (Fig. 5). This result is consistent with Fig. 2B, showing that ΔORF29-iSLK cells undergo viral genome replication.

### The N-terminal region of ORF29 is required for its interaction with ORF7, and full-length ORF29 is required for enhancing the formation of the terminase complex

**Fig 6:**
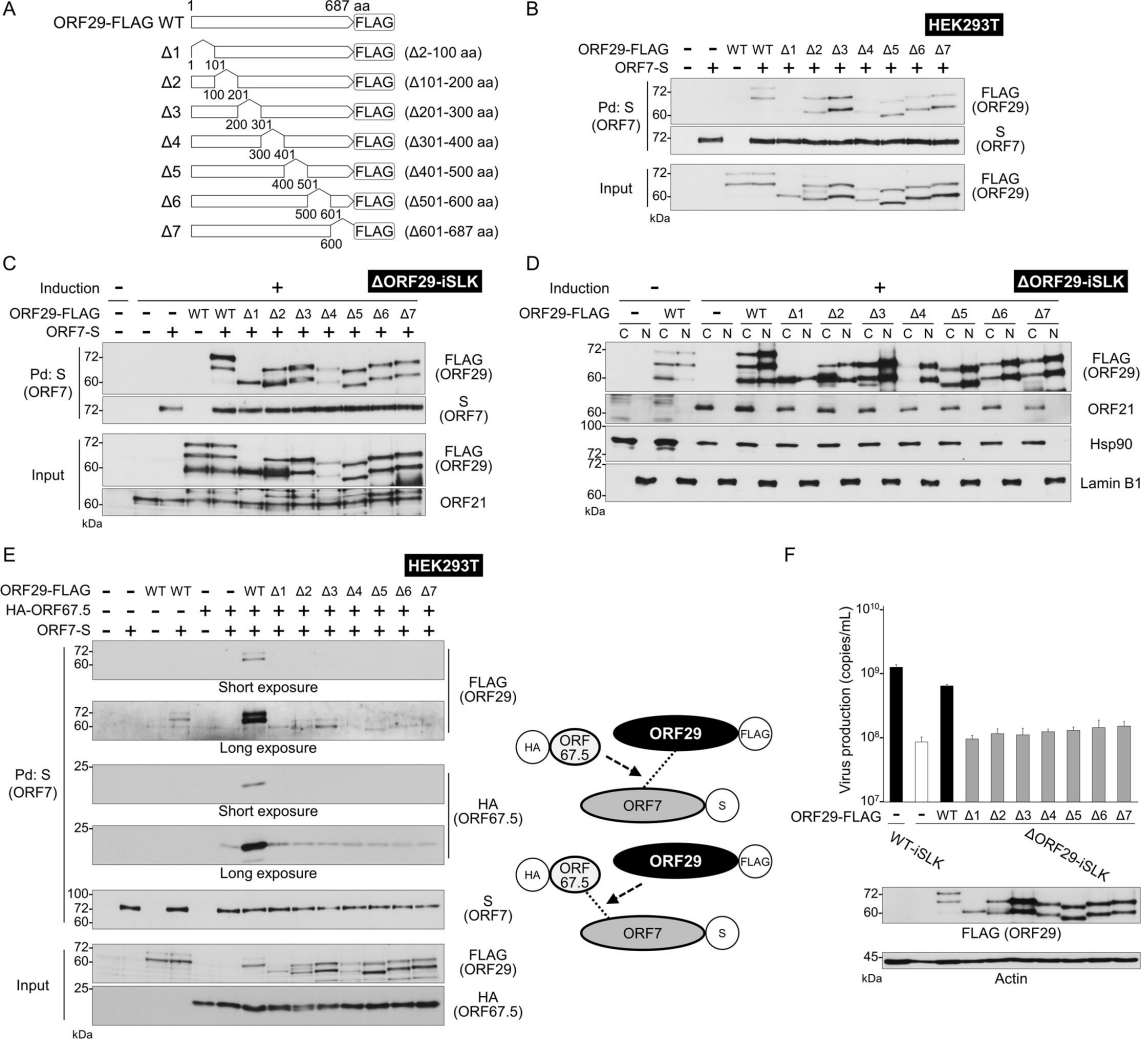
Characterization of exogenous ORF29 deletion mutants. (**A**) Schematic representation of C-terminal FLAG-tagged ORF29 deletion mutants. Deleted amino acids (aa) are shown to the right of each mutant. (**B**) The N-terminal aa 2–100 of ORF29 was essential for its interaction with ORF7. The interaction between ORF29 deletion mutants (Δ1–Δ7) and wild-type (WT) ORF7 was analyzed by pulldown assays. WT ORF7-S plasmid and ORF29-FLAG deletion mutant plasmids were co-transfected into HEK293T cells. Cells were lysed in lysis buffer, and ORF7-S protein was pulled down using S-protein agarose. Precipitates were analyzed by WB. Note: the ORF29 Δ1 mutant lacks the second methionine (M42) of the ORF29. (**C**) ORF7-S plasmid and each ORF29-FLAG deletion mutant plasmid were co-transfected into ΔORF29-iSLK cells. After treatment with Dox and SB, lytic-induced cells were lysed in lysis buffer, and cell extracts were analyzed by pulldown assay. ORF7-S protein was pulled down using S-protein agarose. The expression of endogenous ORF21 protein indicates progression to the lytic reactivation. (**D**) Each ORF29-FLAG deletion mutant plasmid was transfected into ΔORF29-iSLK cells, followed by treatment with Dox and SB for 72 h. Cell extracts were separated into cytosolic [C] and nuclear [N] fractions and analyzed by WB. Hsp90 and Lamin B1 were used as markers for the cytoplasmic and nuclear fractions, respectively. (**E**) All ORF29 deletion mutants lost the ability to enhance the interaction between ORF7 and ORF67.5. Plasmids encoding HA-ORF67.5, ORF7-S, and ORF29-FLAG deletion mutants were co-transfected into HEK293T cells. Cells were lysed in lysis buffer, and ORF7-S protein was pulled down using S-protein agarose. Interaction partners of precipitated ORF7-S were detected by WB. The right panel shows a schematic representation of the tested interaction models. (**F**) Complementation of the reduction in encapsidated viral genome production in ORF29-deficient KSHV cells by ORF29 deletion mutants. ΔORF29-iSLK cells were transfected with respective ORF29 mutant plasmids and cultured with Dox and SB for 72 h to induce the lytic phase. Culture supernatants were collected, and encapsidated viral genome copy numbers were quantified by qPCR. The bottom panel shows a WB using an anti-FLAG Ab to confirm the expression of exogenous ORF29 mutant proteins.

The KSHV terminase complex is thought to comprise ORF7, ORF29, and ORF67.5. These components form a tripartite complex, but ORF29 and ORF67.5 do not interact directly (6). ORF7 interacts with both ORF29 and ORF67.5 and serves as the hub molecule of the tripartite complex (6). To identify the region of ORF29 required for interaction with ORF7, plasmids encoding ORF29 partial deletions (Δ1: Δ2–100 aa, Δ2: Δ101–200 aa, Δ3: Δ201–300 aa, Δ4: Δ301–400 aa, Δ5: Δ301–400 aa, Δ6: Δ501–600 aa, Δ7: Δ601–687 aa) were generated (Fig. 6A). A pulldown assay was performed to test whether each ORF29 deletion mutant could interact with ORF7. The C-terminal S-tagged ORF7 (ORF7-S) plasmid and each C-terminal FLAG-tagged ORF29 mutant (ORF29-FLAG) plasmid were co-transfected into HEK293T cells. Next, the ORF7-S protein was pulled down from the cell lysates using agarose beads conjugated to the S-protein, which binds the S-tag. ORF29 WT and mutants Δ2–7 interacted with ORF7; however, the interaction between ORF29 Δ1 and ORF7 was not detected (Fig. 6B). These results suggest that the N-terminal region of ORF29 (specifically aa 2–100) is important for its interaction with ORF7.

Subsequently, the interaction between ORF7 and the ORF29 deletion mutants was investigated in lytic-induced iSLK cells, where other lytic proteins are present. The ORF7-S plasmid and each ORF29-FLAG plasmid were co-transfected into ΔORF29-iSLK cells. After 24 h, the cells were treated with Dox and SB for 72 h and then subjected to a pulldown assay. Interestingly, all ORF29 deletion mutants interacted with ORF7 (Fig. 6C). Next, we examined the intracellular localization of the ORF29 deletion mutants. Each ORF29-FLAG plasmid was transfected into ΔORF29-iSLK cells, and extracts from lytic-induced cells were fractionated into cytoplasmic and nuclear fractions. As a result, all ORF29 deletion mutants were localized to both the cytoplasm and nucleus (Fig. 6D). ORF21 was localized in the cytoplasm, consistent with our previous report (27).

We previously reported that the interaction between ORF7 and ORF67.5 is enhanced by ORF29, and the interaction between ORF7 and ORF29 is enhanced by ORF67.5 (8). Therefore, we investigated whether the ORF29 deletion mutants could enhance the interaction between ORF7 and ORF67.5. The ORF7-S plasmid, the N-terminal HA-tagged ORF67.5 (HA-ORF67.5) plasmid, and each mutated ORF29-FLAG plasmid were co-transfected into HEK293T cells. Next, the ORF7-S protein was pulled down using S-protein agarose. As expected, the interaction between ORF7 and ORF67.5 was enhanced by ORF29 WT, and the interaction between ORF7 and ORF29 WT was also enhanced by ORF67.5 (Fig. 6E). In contrast to WT ORF29, all ORF29 deletion mutants (ORF29 Δ1–7) failed to enhance the interaction between ORF7 and ORF67.5. Moreover, ORF67.5 did not enhance the interaction between ORF7 and any of the ORF29 Δ1–7 mutants (Fig. 6E). These results show that only full-length ORF29 retains the ability to enhance interactions between the components of the terminase complex. The right panel of Fig. 6E shows a schematic of the interactions of each ORF evaluated in this experiment. Note: the band pattern detected in Fig. 6B (using ORF29-FLAG and ORF7-S) differs from the band pattern in Fig. 6E (using ORF29-FLAG, ORF7-S, and HA-ORF67.5). Because ORF67.5 enhances the binding of ORF29 to ORF7 (8), the band intensities in Fig. 6E differ from those in Fig. 6B.

Finally, the rescue of virus production by the ORF29 deletion mutants was evaluated using a complementation assay. ΔORF29-iSLK cells were transfected with each ORF29 mutant plasmid, and DNase-resistant viral genome copies in the culture supernatant were quantified. Virus production in ΔORF29-iSLK cells was reduced compared to WT-iSLK cells; however, this reduction was rescued by overexpression of ORF29 WT in ΔORF29-iSLK cells (black bars and white bar in Fig. 6F). In contrast, overexpression of all ORF29 deletion mutants (ORF29 Δ1–7) failed to restore virus production (gray bars in Fig. 6F). These results indicate that full-length ORF29 is required for virus production. Specifically, amino acids 2-100 of ORF29 are required for its interaction with ORF7. Moreover, full-length ORF29 is important for enhancing the interactions among the KSHV terminase complex components and for the virus-producing function of ORF29. However, we must consider the possibility that these ORF29 mutants may not retain the native conformation of WT ORF29.

### ORF29 is a self-interacting protein

**Fig 7:**
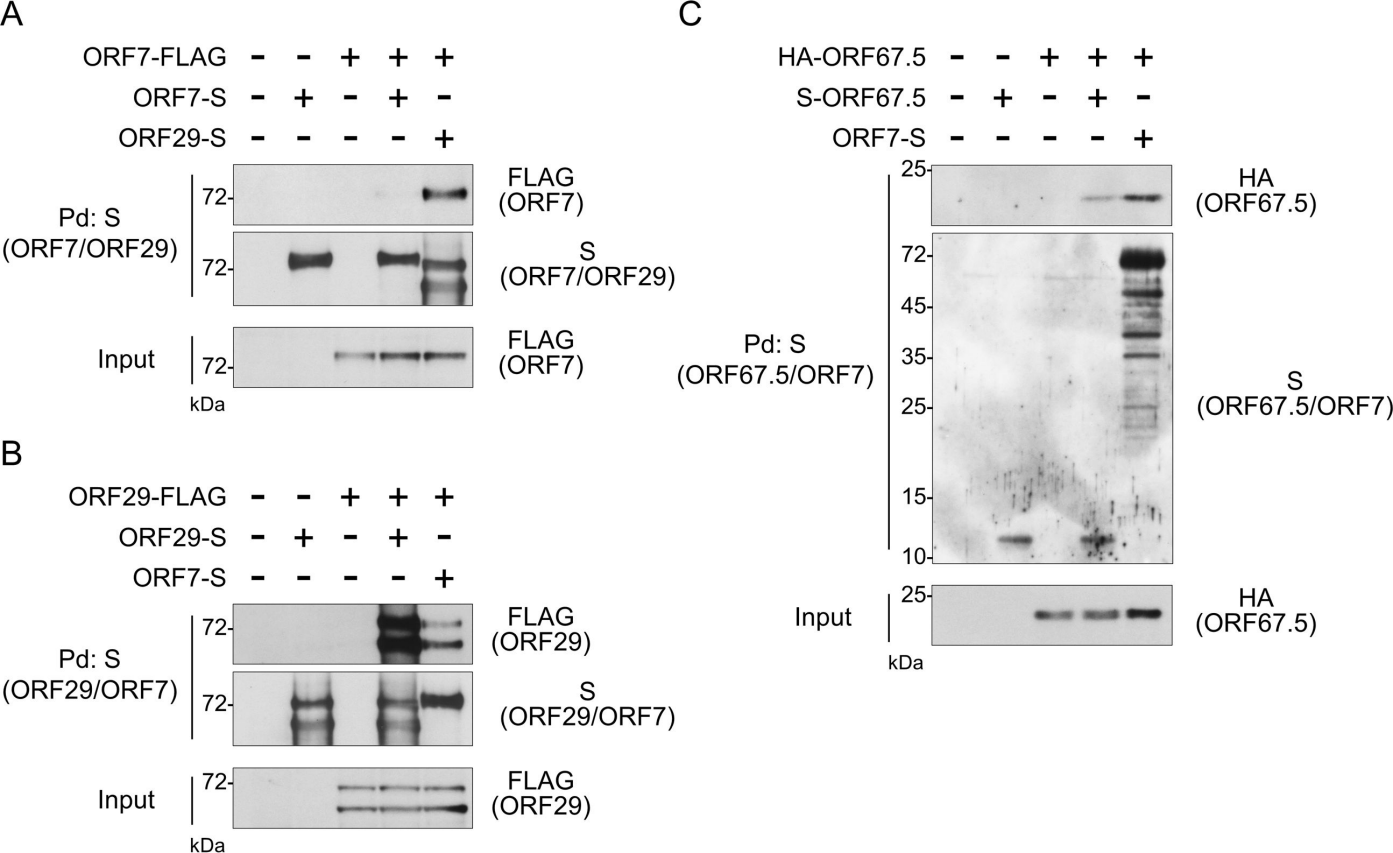
ORF29 preferentially interacted with itself rather than with ORF7. The self-interaction activities of each component of the KSHV terminase complex were evaluated by pulldown assays. Plasmids were co-transfected into HEK293T cells, which were then lysed in lysis buffer. (**A and B**) ORF29-S, (**A‒C**) ORF7-S, or (**C**) S-ORF67.5 proteins were pulled down with S-protein agarose. The precipitated proteins were analyzed by WB using an anti-FLAG Ab to detect (**A and B**) ORF7 and ORF29, or an anti-HA Ab to detect (**C**) ORF67.5.

The KSHV terminase complex consists of ORF7, ORF29, and ORF67.5. The heteromeric protein–protein interactions among the terminase complex components have been characterized. Specifically, ORF7 interacts with both ORF29 and ORF67.5, but ORF29 and ORF67.5 do not interact with one another (6). Previously, it was unknown whether the terminase complex proteins self-interact. Therefore, we examined whether ORF7 is a self-interacting protein. The ORF7-FLAG plasmid and either the ORF7-S or ORF29-S plasmid were co-transfected into HEK293T cells, and the ORF7-S or ORF29-S protein was pulled down with S-protein agarose. The precipitated molecule (i.e., the interacting partner of ORF7-S or ORF29-S) was probed by WB. As expected, the interaction between ORF29-S and ORF7-FLAG was detected. However, we did not detect a self-interaction of ORF7-S-FLAG (Fig. 7A).

Next, the self-interaction of ORF29 was examined. The ORF29-FLAG plasmid and either the ORF29-S or ORF7-S plasmid were co-transfected into HEK293T cells, and the ORF29-S or ORF7-S protein was pulled down to detect its binding partner. As expected, the interaction between ORF7-S and ORF29-FLAG was detected. Interestingly, ORF29-FLAG interacted more strongly with ORF29-S than with ORF7-S (Fig. 7B).

Finally, the self-interaction of ORF67.5 was evaluated. The HA-ORF67.5 plasmid and either the S-ORF67.5 or ORF7-S plasmid were co-transfected into HEK293T cells, and the S-ORF67.5 or ORF7-S protein was pulled down with S-protein agarose. A weaker self-interaction was detected for ORF67.5 compared to its interaction with ORF7 (Fig. 7C). These results demonstrate that ORF29 interacts more strongly with itself than with ORF7; that is, ORF29 is a self-interacting protein.

## DISCUSSION

In this study, we showed that KSHV ORF29 is a component of the terminase complex and plays essential roles in both TR cleavage of viral genome precursors and capsid maturation. Additionally, full-length ORF29 was required to enhance both terminase complex formation and virus production. We also found that ORF29 is a self-interacting protein. To our knowledge, this is the first report demonstrating that ORF29 contributes to terminase function as a component of the KSHV terminase complex. This finding was established by characterization of a fully ORF29-deficient KSHV.

ORF29-deficient KSHV failed to process the viral genome precursor and predominantly formed soccer ball-like capsids in the nuclei of infected cells (Fig. 4B, C, and 5). Similarly, ORF7- or ORF67.5-deficient KSHVs fail to process viral genome precursors and predominantly form soccer ball-like capsids (7, 8). Our previous studies and the results reported here indicate that when KSHV terminase function is impaired, capsid maturation is arrested at an immature stage. The soccer ball-like capsids produced by ORF7-, ORF29-, and ORF67.5-deficient KSHVs share a common internal structure resembling the Telstar pattern or coin dot of a soccer ball (7, 8). It has been hypothesized that when the KSHV genome is assembled into the capsid, the decayed scaffold proteins are extruded and eliminated from the capsid (25). We speculate that the soccer ball-like capsids are immature capsids in which decayed scaffold proteins are not shed but remain inside the capsid (7). As our data show, loss of KSHV terminase function (i.e., failure to process the viral genome precursor) results in the formation of soccer ball-like capsids. This finding supports the hypothesis presented in the literature (25) that decayed scaffold proteins are ejected from the capsid during genome packaging.

The N-terminal region of ORF29 was shown to be important for its interaction with ORF7 (Fig. 6B). An ORF29 deletion mutant lacking amino acids 2–100 showed no interaction with ORF7 (Fig. 6B). Based on these results, the 2–100 amino acid region of ORF29 is important for its interaction with ORF7. In addition to mediating this interaction, the 2–100 amino acid region of ORF29 may also be important for maintaining the proper conformation of ORF29. However, each ORF29 deletion mutant that interacted with ORF7 was unable to enhance the interaction between ORF7 and ORF67.5 (Fig. 6B and E). Based on these results, we hypothesize that each ORF29 deletion mutant may be incapable of forming a functional terminase complex. Taken together, these results suggest that the entire ORF29 protein is required to maintain the proper conformation of the terminase complex. Furthermore, all ORF29-deficient mutants, including the aa 2–100 deletion mutant, interacted with ORF7 in lytic-induced ΔORF29-iSLK cells treated with Dox and SB (Fig. 6C). This suggests that the presence of other KSHV lytic proteins influences the interaction between ORF7 and ORF29.

In a previous study of ORF29, Glaunsinger et al. generated the ORF29.stop virus, which expresses the N-terminal region of ORF29. Their results showed that ORF29 is important for L gene expression and KSHV genome replication (22). However, in our characterization of the ΔORF29-BAC16 clone generated in this study, we observed no impairment in K8.1 (L gene) expression or KSHV genome replication resulting from ORF29 deletion (Fig. 1F, 2A, B, and 5). One possible explanation for the difference between their results and ours may be the difference in the mutation site introduced in the ORF29 gene. Our ΔORF29-BAC16 has a frameshift mutation caused by the deletion of a C–G bp located 1 bp downstream of the ORF29 start codon. This mutation generates a nonsense ORF29 mRNA containing a stop codon 76–78 bp downstream of the start codon and is likely targeted for degradation. Indeed, ΔORF29-BAC16 did not express the ORF29 protein (Fig. 1A and D). In contrast, the ORF29.stop virus used in the previous study was generated by introducing stop codons at the 338th and 339th codons of ORF29 (22). Therefore, it is assumed that the ORF29.stop virus expresses the N-terminal region (aa 1–337) of the full-length ORF29 protein (687 amino acids). This C-terminally truncated ORF29 protein may inhibit L gene expression and KSHV genome replication.

The structure of the KSHV terminase complex has not yet been determined, but the structure of the HSV-1 terminase complex has been resolved by cryo-electron microscopy (28). The components of the HSV-1 terminase complex (UL15, UL28, and UL33) are homologs of KSHV ORF29, ORF7, and ORF67.5, respectively. UL28 interacts with both UL15 and UL33 (29, 30). One molecule each of UL15, UL28, and UL33 forms a tripartite complex, and six of these complexes assemble into a hexameric ring (28). The HSV-1 genome passes through this terminase ring during genome packaging into capsids (28). In this study, KSHV ORF29 was found to be a strongly self-interacting protein (Fig. 7B), while KSHV ORF67.5 exhibited weaker self-interaction. The self-interaction of ORF29 (and ORF67.5) may contribute to the multimerization of the terminase complex or to the formation of the terminase ring through self-assembly of multiple ORF29 molecules. The significance of ORF29’s self-interaction remains unknown and requires further investigation.

We have focused on the KSHV terminase complex and characterized its components. Currently, ORF7, ORF29, and ORF67.5 are considered components of the KSHV terminase complex. Our previous studies have shown that ORF7 and ORF67.5 are important for KSHV terminase function (6–8). This study showed that ORF29 is also important for KSHV terminase function. Thus, we have advanced the functional characterization of the KSHV terminase complex. The herpesvirus terminase machinery is essential for viral replication but is not present in humans. Therefore, the KSHV terminase complex is a promising target for anti-KSHV drug development. Additionally, elucidation of the currently unresolved structure of the KSHV terminase complex will deepen insight into its function and facilitate the development of anti-KSHV drugs.

## MATERIALS AND METHODS

### Cell culture and reagents

HEK293T cells were cultured in Dulbecco’s modified Eagle’s medium (DMEM) (Nacalai Tesque Inc., Kyoto, Japan) supplemented with 10% fetal bovine serum (FBS). iSLK cells (23) were cultured in DMEM supplemented with 10% FBS, 1 µg/mL of puromycin (InvivoGen, CA, USA), and 0.25 mg/mL of G418 (Nacalai Tesque, Inc.). iSLK cells harboring KSHV-BAC were cultured in DMEM supplemented with 10% FBS, 1 µg/mL of puromycin (InvivoGen), 0.25 mg/mL of G418 (Nacalai Tesque, Inc.), and 1 mg/mL of hygromycin B (Wako, Osaka, Japan).

### Plasmids

The C-terminal 3× FLAG-tagged ORF29 expression plasmid (RF-009), the C-terminal 2× S-tagged ORF7 expression plasmid (YI-02), the C-terminal 3× FLAG-tagged ORF7 expression plasmid (YI-04), the N-terminal 2× S-tagged ORF67.5 expression plasmid (YI-16), and the N-terminal 5× HA-tagged ORF67.5 expression plasmid (YI-17) have been described previously (6, 8). The C-terminal 2× S-tagged ORF29 expression plasmid (RF-010) was constructed by PCR using the previously constructed N-terminal 3× FLAG-tagged ORF29 expression plasmid (YI-52) as a template and digesting the obtained insert with EcoRI (Takara Bio, Shiga, Japan) and SalI (TOYOBO, Osaka, Japan) (6). The DNA ligation kit Mighty Mix (Takara Bio) was used for ligation. Each C-terminal 3× FLAG-tagged ORF29 mutant expression plasmid (Δ1 [YI-94], Δ2 [YI-95], Δ3 [YI-96], Δ4 [YI-97], Δ5 [YI-98], Δ6 [YI-99], Δ7 [YI-100]) was constructed using the In-Fusion HD Cloning kit (Takara Bio). The inserts were obtained by PCR using the C-terminal 3× FLAG-tagged ORF29 expression plasmid (RF-009) as a template. The KOD-Plus-Neo (TOYOBO) was used for PCR, and the pCI-neo mammalian expression vector (Promega, WI, USA) was used as the backbone vector. The primers used for plasmid construction are listed in Table 1. The sequences of the inserts were confirmed by Sanger sequencing.

**TABLE 1:** Primers for BAC mutagenesis, construction of plasmids, and qPCR

Primer name	Primer sequences (5′ → 3′)
BAC mutagenesis	
Fw_dORF29_EP	TGCAGGTTGGCGGCAAGGCGCTCCCTGTGACGGCTGAGCACATGTTTGCTTTGAGCTCGCTAGGGATAACAGGGTAATCGATTT
Rv_dORF29_EP	CGGGTCACCCTCGGACACGAGCGAGCTCAAAGCAAACATGTGCTCAGCCGTCACAGGGAGGCCAGTGTTACAACCAATTAACC
Fw_dORF29_REV_EP	TGCAGGTTGGCGGCAAGGCGCTCCCTGTGACGGCTGAGCAGCATGTTTGCTTTGAGCTCGCTAGGGATAACAGGGTAATCGATTT
Rv_dORF29_REV_EP	CGGGTCACCCTCGGACACGAGCGAGCTCAAAGCAAACATGCTGCTCAGCCGTCACAGGGAGGCCAGTGTTACAACCAATTAACC
Construction plasmids	
Fw_EcoRI_ORF29	CATGAATTCATGCTGCTCAGCCGTCACAG
Rv_SalI_ORF29_C	CAAGTCGACTTGTGGGGATATGGGCTTGTAC
Fw_ORF29_ifu	CTAGCCTCGAGAATTCATGCTGCTCAGCCGTCA
Fw_ORF29_d1_ifu	CTAGCCTCGAGAATTCATGACATACTTGCAGATGC
Fw_ORF29_d2	CGCGCCTATTGGAGTCCCTATCATTATTTGAAACTCCAAACAGTGTTTTACAG
Rv_ORF29_d2	CTGTAAAACACTGTTTGGAGTTTCAAATAATGATAGGGACTCCAATAGGCGCG
Fw_ORF29_d3	GGATTGTTTAAGCAGTACTTCGGTGCAACATGCTTCAATAAGAACAGC
Rv_ORF29_d3	GCTGTTCTTATTGAAGCATGTTGCACCGAAGTACTGCTTAAACAATCC
Fw_ORF29_d4	CCAGCTCGCTGATGTGCATTCCGACGTACATCAC
Rv_ORF29_d4	GTGATGTACGTCGGAATGCACATCAGCGAGCTGG
Fw_ORF29_d5	CTGTCCTTGTTACAGACTGCACACCAGAAGCCTCATATTGGG
Rv_ORF29_d5	CCCAATATGAGGCTTCTGGTGTGCAGTCTGTAACAAGGACAG
Fw_ORF29_d6	GTCACGAGTACTCAGACTCCCGAGACATTCATCTACGCTCTG
Rv_ORF29_d6	CAGAGCGTAGATGAATGTCTCGGGAGTCTGAGTACTCGTGAC
Rv_ORF29_d7_ifu	TACCACGCGTGAATTCAAACGCGGAGGATTTCTC
Rv_ORF29_ifu	TACCACGCGTGAATTCTTGTGGGGATATGGGCT
qPCR	
qPCR_KSHV_ORF11-Fw	TTGACAACACGCACCGCAAG
qPCR_KSHV_ORF11-Rv	AAAAATCAGCACGCTCGAGGAG
Fw_GAPDH_RTqPCR	CATCAAGAAGGTGGTGAAGCAG
Rv_GAPDH_RTqPCR	TGTCGCTGTTGAAGTCAGAGG
Fw_ORF16_RTqPCR	AGATTTCACAGCACCACCGGTA
Rv_ORF16_RTqPCR	CCCCAGTTCATGTTTCCATCGC
Fw_ORF46/47_RTqPCR	CGATCCGAATCACTGCAACG
Rv_ORF46/47_RTqPCR	CTGCTGCTTTTAGCCCGAG
Fw_K8.1_RTqPCR	TCCCACGTATCGTTCGCATTTGG
Rv_K8.1_RTqPCR	GCGTCTCTTCCTCTAGTCGTTG
Fw_ORF7_RTqPCR	TTTATTTCCCAGTCCTCCAAATG
Rv_ORF7_RTqPCR	GGGAAGCATGCCCGC
Fw_ORF17_RTqPCR	AGTGGGTGGTTTCCAGATTCTC
Rv_ORF17_RTqPCR	GGACTGACGAAATTTGGTGTGG
Fw_ORF675_RTqPCR	TGCAGCCTGCGATCATACTC
Rv_ORF675_RTqPCR	AATACGGCGTCCGTGCTC

### Mutagenesis of BAC16 clones

Mutagenesis of BAC16 clones was performed as described in previous publications (24, 31). ΔORF29-BAC16 was constructed by deleting a single G at position 54,489 in WT-BAC16 (accession number: GQ994935). Revertant-BAC16 was generated by reinsertion of the G missing in ΔORF29-BAC16. The primers used for this mutagenesis are shown in Table 1. The insertion and deletion of kanamycin resistance cassettes in each BAC16 clone were analyzed by EcoRI or SalI digestion and agarose gel electrophoresis. The mutated sites of each BAC16 clone were verified by Sanger sequencing.

### Generation of iSLK cells stably harboring individual BAC16 clones

Each BAC16 clone was purified from the *Escherichia coli* strain GS1783 by NucleoBond Xtra BAC (Takara Bio). WT-BAC16, ΔORF29-BAC16, and Revertant-BAC16 were transfected into iSLK cells by the calcium phosphate method. The transfected iSLK cells were selected under 1  mg/mL of hygromycin B (Wako) to establish cell lines stably harboring Dox-inducible recombinant KSHV (WT-iSLK, ΔORF29-iSLK, and Revertant-iSLK).

### Measurement of viral gene expression, intracellular viral genome replication, and virus production

Each measurement was performed according to previously described methods with slight modifications (8). Briefly, iSLK cells harboring WT or each BAC16 mutant were treated with 8  µg/mL of Dox and 1.5 mM of SB for 72 h to induce lytic reactivation.

To measure viral gene expression, lytic-induced or uninduced cells (3.5 × 10^5^ cells in a 6-well plate) were harvested with 500 µL of RNAiso Plus (Takara Bio). The extracted total RNA was treated with DNase I (New England Biolabs, MA, USA) and resuspended in 300 µL of RNAiso Plus (Takara Bio). The cDNA was synthesized from 160 ng of DNase-treated total RNA using ReverTra Ace qPCR RT Master Mix (TOYOBO). qPCR was performed using the synthesized cDNA as a template with the THUNDERBIRD Next SYBR qPCR mix (TOYOBO). Table 1 lists the primers used to measure viral gene expression. The relative mRNA expression levels were determined by the delta-delta threshold cycle (ΔΔCT) method and were normalized to GAPDH mRNA levels.

To quantify intracellular viral genome replication, iSLK cells (3.5 × 10^4^ cells in a 48-well plate) were induced or uninduced and harvested. Viral genome DNA and cellular genomic DNA were purified from the harvested cells using a QIAamp DNA Blood mini kit (Qiagen, CA, USA). The viral genome copy number was quantified by qPCR and normalized to the amount of total DNA. qPCR assays were performed using the THUNDERBIRD Next SYBR qPCR mix (TOYOBO) and the KSHV ORF11-specific primers, which are listed in Table 1.

To quantify extracellular encapsidated viral DNA, iSLK cells (1.5 × 10^5^ cells in a 12-well plate) were induced, and culture supernatants were harvested and centrifuged to remove debris. The supernatants were treated with DNase I (New England Biolabs), and encapsidated viral DNA was extracted from the supernatants using a QIAamp DNA Blood mini kit (Qiagen). Purified viral DNA copy numbers were quantified by qPCR using the THUNDERBIRD Next SYBR qPCR mix (TOYOBO) and the KSHV ORF11-specific primers.

Infectious virus production was quantified using a supernatant transfer assay. iSLK cells (2 × 10^6^ cells on a 10 cm dish) were induced, and the culture supernatants and cells were collected. The supernatants and cells were centrifuged, and the supernatants were mixed with fresh HEK293T cells (7.5 × 10^5^ cells) and polybrene (8 µg/mL; Sigma-Aldrich, MO, USA). The mixtures were added to 12-well plates. The plates were centrifuged at 1,200 × *g* for 2 h and incubated for 24 h. GFP-positive cells were detected with a flow cytometer (FACSCalibur, Becton Dickinson, NJ, USA) using CellQuest Pro software (Becton Dickinson).

### Complementation assay

iSLK cells (1.5 × 10^5^ cells in a 12-well plate for quantification of extracellular encapsidated viral DNA, or 2 × 10^6^ cells on a 10 cm dish for measurement of infectious virus production) were induced with 8 µg/mL of Dox and 1.5 mM of SB and concurrently transfected with each plasmid. After 72 h, the respective evaluations were conducted according to the methods described in the section entitled “Measurement of viral gene expression, intracellular viral genome replication, and virus production.”

### Western blotting

Cells were washed with phosphate-buffered saline (PBS) and lysed in SDS sample buffer (50 mM Tris-HCl [pH 6.8], 5% [wt/vol] SDS, 50% [vol/vol] glycerol, 0.002% [wt/vol] bromophenol blue, and 2% [vol/vol] 2-mercaptoethanol). Next, the samples were sonicated for 10 s, reduced at 60°C for 20 min, and subjected to SDS-PAGE. The ExcelBand All Blue Regular Range Protein Marker (PM1500) (SMOBIO, Hsinchu County, Taiwan) was used as an Mw marker. The proteins were transferred to a ClearTrans nitrocellulose membrane 0.2 µm (Wako), and the membrane was incubated for 30 min at room temperature in 5% (wt/vol) nonfat dry milk in PBS with 0.1% (vol/vol) Tween-20 (PBS-T). The membrane was then incubated with a primary Ab, followed by incubation with a secondary Ab in Can Get Signal Immunoreaction Enhancer Solution (TOYOBO). Immunodetection was achieved with the ECL Western Blotting Detection Reagents (Cytiva, Tokyo, Japan). The blot was then exposed to X-ray film (Fuji film, Tokyo, Japan).

### Antibodies

Anti-KSHV ORF29 rabbit pAb was generated by GL Biochem, Shanghai, China, using the synthetic peptide GERWELSAPTFTRHCPKTAR (ORF29: aa 22 to 41) as the antigen. Anti-KSHV ORF29 rabbit pAb was purified from the immunized rabbit serum using antigen peptide affinity chromatography. The following primary Abs were used: anti-ORF45 mouse monoclonal Ab (mAb; 2D4A5; Santa Cruz Biotechnology, TX, USA), anti-K-bZIP mouse mAb (F33P1; Santa Cruz Biotechnology), anti-K8.1 A/B mouse mAb (4A4; Santa Cruz Biotechnology), anti-ORF21 rabbit pAb (previously produced in our laboratory) (27), anti-beta actin mouse mAb (AC-15; Santa Cruz Biotechnology), anti-FLAG-tag mouse mAb (FLA-1; MBL, Nagoya, Japan), anti-S-tag rabbit pAb (sc-802; Santa Cruz Biotechnology), anti-HA-tag mouse mAb (TANA2; MBL), anti-Hsp90 mouse mAb (68/Hsp90; Becton Dickinson), and anti-Lamin B1 rabbit mAb (D4Q4Z; Cell Signaling Technology, MA, USA). Anti-mouse IgG-horseradish peroxidase-conjugated (HRP; NA931; Cytiva) and anti-rabbit IgG-HRP (7074; Cell Signaling Technology) were used as the secondary Abs.

### Subcellular fractionation

iSLK cells (3.5 × 10^5^ cells in a 6-well plate) were transfected with 4 µg of plasmid DNA and 12 µg of polyethylenimine hydrochloride (PEI) MAX (Polysciences, Inc., PA, USA) for 24 h. The cells were then induced with 8 µg/mL of Dox and 1.5 mM of SB. After 72 h, cells were incubated on ice for 15 min with 100 µL of hypotonic buffer containing 10 mM HEPES (pH 8.0), 10 mM KCl, 0.1 mM EDTA, 0.1 mM EGTA, and 1 mM dithiothreitol. Cells were lysed by the addition of Nonidet P-40 substitute to a final concentration of 0.625%, followed by vortex agitation. Cell lysates were centrifuged at 15,000 rpm for 30 s at 4°C, and the supernatant was collected as the cytoplasmic fraction. The nuclear pellets were washed three times with hypotonic buffer supplemented with 0.625% Nonidet P-40 substitute. The cytoplasmic and nuclear fractions were resuspended in SDS-PAGE sample buffer and subjected to WB. Hsp90 and Lamin B1 were used as markers for the cytoplasmic and nuclear fractions, respectively.

### Immunoprecipitation and pulldown assay

Ten 10 cm dishes of each iSLK cell line harboring BAC (2 × 10^6^ cells/dish) were induced or uninduced for 72 h. HEK293T cells (2 × 10^6^ cells on a 10 cm dish) were transfected with 12 µg of plasmid DNA and 36 µg of PEI MAX (Polysciences) for 20 h. The cells were lysed in lysis buffer (50 mM Tris-HCl [pH 8.0], 120 mM NaCl, 1% [vol/vol] glycerol, 0.2% [vol/vol] Nonidet P-40 substitute, and 1 mM dithiothreitol) or RadioImmunoPrecipitation Assay (RIPA) buffer (50 mM Tris-HCl [pH 8.0], 150 mM NaCl, 1% [vol/vol] Nonidet P-40 substitute, 0.5% [wt/vol] sodium deoxycholate, and 0.1% [wt/vol] SDS). The buffers used for lysis are described in each figure legend. The cell extracts were incubated with appropriate beads for 1 h, and the beads were washed three times. S-protein agarose (Merck KGaA, Darmstadt, Germany) and Dynabeads Protein G (Thermo Fisher Scientific, MA, USA) were used in this study. The beads used are described in each figure legend. The washed beads were resuspended in SDS sample buffer and incubated at 95°C for 10 min. The precipitates were detected by WB.

### Electron microscopy and Southern blotting

Electron microscopy and Southern blotting were performed according to previously described methods (8).

### Statistics

The statistical significance was determined by one-way analysis of variance followed by a Dunnett’s test and was evaluated using GraphPad Prism 7 software (GraphPad Software, CA, USA).

## Data Availability

The underlying data and accession numbers are available in the main text. All other raw data are available upon request.
